# Accumulation of Dense Core Vesicles in Hippocampal Synapses Following Chronic Inactivity

**DOI:** 10.3389/fnana.2018.00048

**Published:** 2018-06-11

**Authors:** Chang-Lu Tao, Yun-Tao Liu, Z. Hong Zhou, Pak-Ming Lau, Guo-Qiang Bi

**Affiliations:** ^1^Center for Integrative Imaging, National Laboratory for Physical Sciences at the Microscale, University of Science and Technology of China, Hefei, China; ^2^School of Life Sciences, University of Science and Technology of China, Hefei, China; ^3^CAS Key Laboratory of Brain Function and Disease, University of Science and Technology of China, Hefei, China; ^4^The California NanoSystems Institute, University of California, Los Angeles, Los Angeles, CA, United States; ^5^Department of Microbiology, Immunology and Molecular Genetics, University of California, Los Angeles, Los Angeles, CA, United States; ^6^CAS Center for Excellence in Brain Science and Intelligence Technology, University of Science and Technology of China, Hefei, China

**Keywords:** synaptic structure, dense core vesicle, homeostatic plasticity, cryo-electron tomography, tetrodotoxin

## Abstract

The morphology and function of neuronal synapses are regulated by neural activity, as manifested in activity-dependent synapse maturation and various forms of synaptic plasticity. Here we employed cryo-electron tomography (cryo-ET) to visualize synaptic ultrastructure in cultured hippocampal neurons and investigated changes in subcellular features in response to chronic inactivity, a paradigm often used for the induction of homeostatic synaptic plasticity. We observed a more than 2-fold increase in the mean number of dense core vesicles (DCVs) in the presynaptic compartment of excitatory synapses and an almost 20-fold increase in the number of DCVs in the presynaptic compartment of inhibitory synapses after 2 days treatment with the voltage-gated sodium channel blocker tetrodotoxin (TTX). Short-term treatment with TTX and the N-methyl-D-aspartate receptor (NMDAR) antagonist amino-5-phosphonovaleric acid (AP5) caused a 3-fold increase in the number of DCVs within 100 nm of the active zone area in excitatory synapses but had no significant effects on the overall number of DCVs. In contrast, there were very few DCVs in the postsynaptic compartments of both synapse types under all conditions. These results are consistent with a role for presynaptic DCVs in activity-dependent synapse maturation. We speculate that these accumulated DCVs can be released upon reactivation and may contribute to homeostatic metaplasticity.

## Introduction

Neural activity can profoundly shape the structure and function of synapses and plays crucial roles in the construction and reconfiguration of neuronal circuits in the brain (Goodman and Shatz, 1993; Zito and Svoboda, 2002; Dan and Poo, 2004; Espinosa and Stryker, 2012). It has long been known that synaptic formation and maturation may be facilitated by neuronal activity (Cohen-Cory, 2002; Andreae and Burrone, 2014). In neocortical explant cultures, chronic treatment with tetrodotoxin (TTX) that blocks neuronal action potentials caused reduction in the number of synapses and nerve terminals with synaptic vesicles, as well as decreased pre- and postsynaptic membrane-associated densities in electron micrographs (Janka and Jones, 1982). In other systems, neuronal activity participates in the process of synaptic formation through various signaling mechanisms (Andreae and Burrone, 2014). Some of these mechanisms, e.g., release of brain-derived neurotrophic factor (BDNF) and calcium influx through activation of the N-methyl-D-aspartate receptor (NMDAR), are also used in the induction of Hebbian synaptic plasticity in more mature synapses, such as long-term potentiation (LTP) and spike-timing-dependent plasticity (STDP; Bi and Poo, 1998; Constantine-Paton and Cline, 1998; Ying et al., 2002; Malenka and Bear, 2004). Such mechanisms involve a positive feedback process: neuronal or synaptic activation causes synaptic strengthening, which in turn leads to higher levels of activity (Bi and Poo, 2001; Fauth and Tetzlaff, 2016).

Complementary to the positive feedback in activity-driven synapse formation and Hebbian plasticity is homeostatic plasticity, in which changes in the overall level of neuronal activity can cause compensatory up- or down-regulation of synaptic function (Turrigiano and Nelson, 2004; Kaneko and Stryker, 2017), thus maintaining the proper activity set point for neurons and networks (Turrigiano, 2008). Typically, chronic blockade of neuronal excitation with TTX or excitatory synaptic transmission with 2,3-dihydroxy-6-nitro-7-sulfamoyl-benzo[f]quinoxaline-2,3-dione (NBQX) leads to increased synapse size and upscaling of synaptic efficacy (Turrigiano et al., 1998; Murthy et al., 2001; Gainey et al., 2009, 2015). Likewise, short-term blockade of neuronal activity with TTX together with the NMDAR antagonist amino-5-phosphonovaleric acid (AP5) could induce rapid upscaling of synaptic strength (Sutton et al., 2006). Beyond synaptic scaling, another form of synaptic homeostasis is metaplasticity (Abraham and Bear, 1996; Abraham and Tate, 1997), which involves adaptive changes in the ability of the synapse to undergo activity induced plasticity such as LTP or LTD, rather than direct changes in the efficacy of synaptic transmission (Thiagarajan et al., 2005). Electrophysiological studies showed that chronic inactivation of neuronal networks not only induced upscaling of synaptic quantal content, but also enhanced subsequent LTP (Arendt et al., 2013; Gerkin et al., 2013).

Structurally, the formation of a synapse as well as its strengthening requires building blocks. For example, α-amino-3-hydroxy-5-methyl-4-isoxazolepropionic acid receptors (AMPARs) are known to be incorporated into the postsynaptic membrane in both Hebbian and homeostatic plasticity (Vitureira and Goda, 2013). On the presynaptic side, dense core vesicles (DCVs) contain the components of the presynaptic active zone and can assemble the presynaptic active zone by fusion with the presynaptic plasma membrane (Zhai et al., 2001; Shapira et al., 2003; Ziv and Garner, 2004). It was also shown that cultured hippocampal slices at early recovery stages exhibited elevated turnover of DCVs, suggesting their role in synaptogenesis (Sorra et al., 2006). Interestingly, presynaptic BDNF, presumably also stored in DCVs, appears to be involved in a presynaptic component of LTP in the hippocampus (Zakharenko et al., 2003; Bekinschtein et al., 2008; Dieni et al., 2012).

In the present study, we use cryo-electron tomography (cryo-ET) to visualize subcellular 3D structures in excitatory and inhibitory synapses of cultured hippocampal neurons in their native state (Tao et al., 2012, 2018). With this method, we investigate structural changes in synapses induced by activity blockade over different periods of time, focusing on the accumulation of presynaptic DCVs.

## Materials and Methods

All animal experiments were approved and conducted according to protocols approved by the Animal Experiments Committee at the University of Science and Technology of China.

### Primary Culture of Hippocampal Neurons

Low-density cultures of dissociated embryonic rat hippocampal neurons were grown on EM grids as previously described (Tao et al., 2018). Briefly, Quantifoil R2/2 gold EM grids with holey carbon film were cleaned using a plasma cleaning system (Gatan), and sterilized with UV light. These grids were then coated with poly-L-lysine (Sigma) before being used for culture. Hippocampi were isolated from embryonic day-18 rats and treated with trypsin followed by gentle trituration to obtain dissociated cells, which were plated on the poly-L-lysine coated EM grids in 35-mm Petri dishes at a density of 40,000–60,000 cells/ml and maintained in incubators at 37°C in 5% CO_2_. The culture medium was NeuroBasal (Invitrogen) supplemented with 5% heat-inactivated bovine calf serum (PAA) plus 5% heat-inactivated fetal bovine serum (HyClone), 1× Glutamax (Invitrogen) and 1× B27 (Invitrogen). To maintain the culture, half of the original medium was replaced by serum-free culture medium 24 h after plating, and one third of the culture medium was replaced with fresh serum-free medium twice a week. The cultures were treated with cytosine arabinoside (Sigma) at various stages to prevent overgrowth of glial cell.

For chronic inactivation, 1 μM TTX was added to the culture medium 2 days prior to cryo-fixation, typically at 16 days *in vitro* (DIV). For short-term inactivation, the cultures were treated with 2 μM TTX for 4 h, with 50 μM AP5 present in the last 3 h prior to cryo-fixation, similar to a previously-established paradigm (Sutton et al., 2006). In control groups, equal volume of culture medium was added without TTX or AP5.

### Frozen-Hydrated Sample Preparation

Low-density neuronal cultures (16 DIV) grown on EM grids taken from the culture incubator were first placed in extracellular solution (ECS, containing 150 mM NaCl, 3 mM KCl, 3 mM CaCl_2_, 2 mM MgCl_2_, 10 mM HEPES and 5 mM glucose, pH 7.3), then mounted on a Vitrobot IV (FEI). For experimental groups, the same concentration of TTX and AP5 was added in the ECS. Protein A-coated colloidal gold beads (15-nm size, CMC) were added to the grid (4 μl each, stock solution washed in ECS and diluted 10 times after centrifugation) as fiducial markers. The grids were then plunged into liquid ethane for rapid vitrification. The samples were then stored in liquid nitrogen until use.

### Cryo-ET Data Collection

Cryo-ET data were collected with single-axis tilt using either a Tecnai F20 transmission electron microscope (TF20, FEI) equipped with an Eagle 4K×4K multiport CCD camera (FEI), or a Titan Krios (FEI) with a K2 Summit direct electron detector (K2 camera, Gatan). The TF20 was operated at an acceleration voltage of 200 KV, with tilt series collected from −60° to +60° at 2° intervals using FEI *Xplore 3D* software. Defocus values of −12 to −18 μm and total electron dosage of ~100 e^−^/Å^2^ were used for all imaging with TF20, with final pixel size of 0.755 nm. The Titan Krios was operated at an acceleration voltage of 300 KV. Images were collected by the K2 camera in counting mode, with tilt series acquired from −64° to +64° at 2° intervals using *Leginon* (Suloway et al., 2005). A defocus value of −10 μm and total accumulated dose of ~120 e^−^/Å^2^ was used for all imaging with Titan Krios, with final pixel size of 0.765 nm.

### 3D Reconstruction and Rendering

Tilt series were aligned and reconstructed using *IMOD* (Kremer et al., 1996). Fifteen nanometer gold beads were used as fiducial markers to align the tilt series. Reconstruction was performed using a simultaneous iterative reconstruction technique with 5 or 15 iterations.

For each synapse, a domain of presynaptic membrane opposing the postsynaptic membrane with a uniformly widened cleft was identified as a contact area. We think this contact area is equivalent to the “active zone” area observed in classical EM studies (Südhof, 2012). This area was manually segmented using *3dmod* in *IMOD* software and triangulated using “*imodmesh*” to make surface. DCVs were identified by visual inspection and manually segmented as spheres using *3dmod*. The segmented structures of presynaptic membrane and DCVs were converted to *vrml* file using the function “*imod2vrml*” in IMOD package. SVs were identified using template-matching as described below and rendered as spheres. The segmented structures of presynaptic membrane, DCVs, and SVs were visualized and presented using *UCSF Chimera* (RRID: SCR_004097, Pettersen et al., 2004).

### Quantitative Analyses and Measurements

To segment SVs, a set of featureless spherical shells of 5 nm thickness with diameter ranging from 25 nm to 70 nm at 1 nm-interval were designed as templates. These templates were Gaussian low-pass filtered to 10 nm resolution with *EMAN2.1*, and used for template matching of SVs in the tomograms using *PyTom* (Hrabe et al., 2012). The results of template matching were evaluated by visual inspection, and mismatches were discarded.

The “*lasso*” tool in *3dmod* was used to measure the maximum sectional area *A* of each DCV in the XY plane. The diameter *d* of a DCV was estimated as $d\;=\;2\sqrt{\left(A/\pi \right)}$ . For the measurement of luminal densities of SVs and DCVs, tomogram density of pixels inside the 3D structure of a SV or DCV were averaged to obtain raw density. To compensate for variable imaging conditions in different tomograms, the raw density was normalized by subtracting the mean raw density of all regular vesicles in that tomogram and then dividing by the standard variation of raw densities of these regular vesicles.

To measure the distance of a DCV to the active zone area of the corresponding synapse, we first calculated the distances from the center of a DCV to all the triangles that make up the surface of the segmented active zone membrane. The distance of the DCV to the active zone was defined as the minimum of these distances minus the radius of the DCV.

### Statistics

All error bars in the figures are standard error of the mean (SEM). All measurements are presented in the text as mean ± SEM except for cases otherwise noted. The statistical tests, which were used to determine the statistical difference between samples, are indicated in “Results” section. For all statistical tests, *p* value < 0.05 was considered statistically significant.

## Results

### Synaptic Dense Core Vesicles in Hippocampal Culture Visualized by Cryo-ET

**Figure 1:**
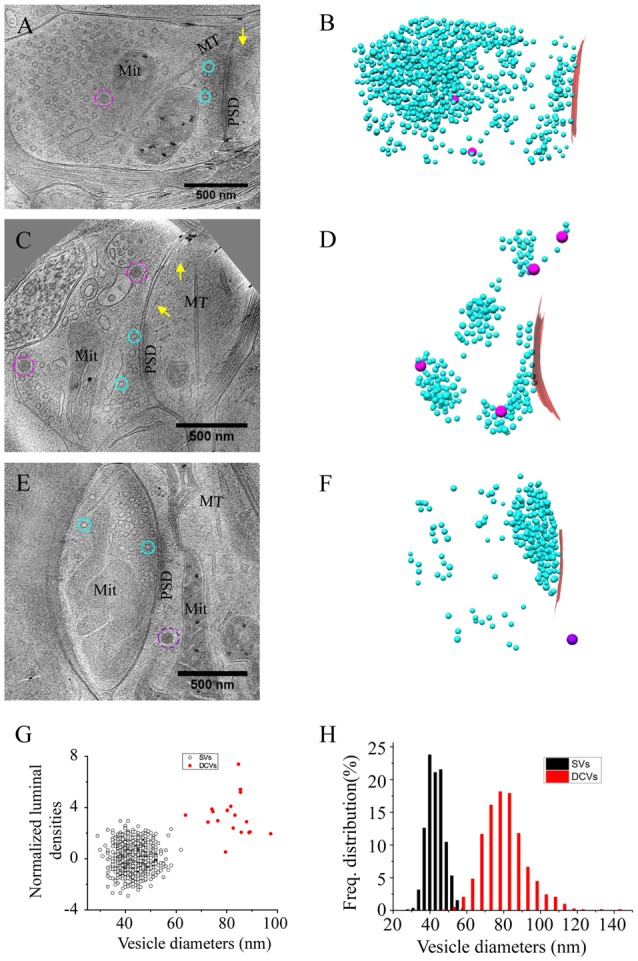
Ultrastructure of excitatory synapses in hippocampal culture revealed by cryo-electron tomography (cryo-ET). **(A,C,E)** Three tomographic slices showing the structures of excitatory synapses formed on spine **(A)** and dendritic shafts **(C,E)**. Subcellular structures, such as SVs (cyan circles), presynaptic (magenta circles) and postsynaptic (purple circle) dense core vesicles (DCVs), actin filaments (yellow arrows), mitochondria (Mit), microtubules (MT) and postsynaptic density (PSD) are clearly visible. **(B,D,F)** 3D rendering of SVs (cyan ball), presynaptic DCVs (magenta balls), postsynaptic DCV (purple ball), and presynaptic membrane of active zone area (red) in the three synapses shown in **(A,C,E)**. In the synapses shown in **(A,C)**, there are two and four DCVs in the presynaptic boutons, respectively. Neither synapse has DCVs in the postsynaptic compartment. In the synapse shown in **(E)**, there is no DCV in the presynaptic bouton but 1 DCV in the postsynaptic compartment. **(G)** Scatter plot of normalized luminal density against the size of 932 SVs and 18 DCVs from four synapses shows two well-defined clusters. **(H)** Histogram shows the distribution of diameters of DCVs (Mean ± SD, 80.1 ± 11.9 nm, *n* = 833 from 185 synapses) and SVs (Mean ± SD, 43.1 ± 4.6 nm, *n* = 932 from 4 synapses).

**Figure 2:**
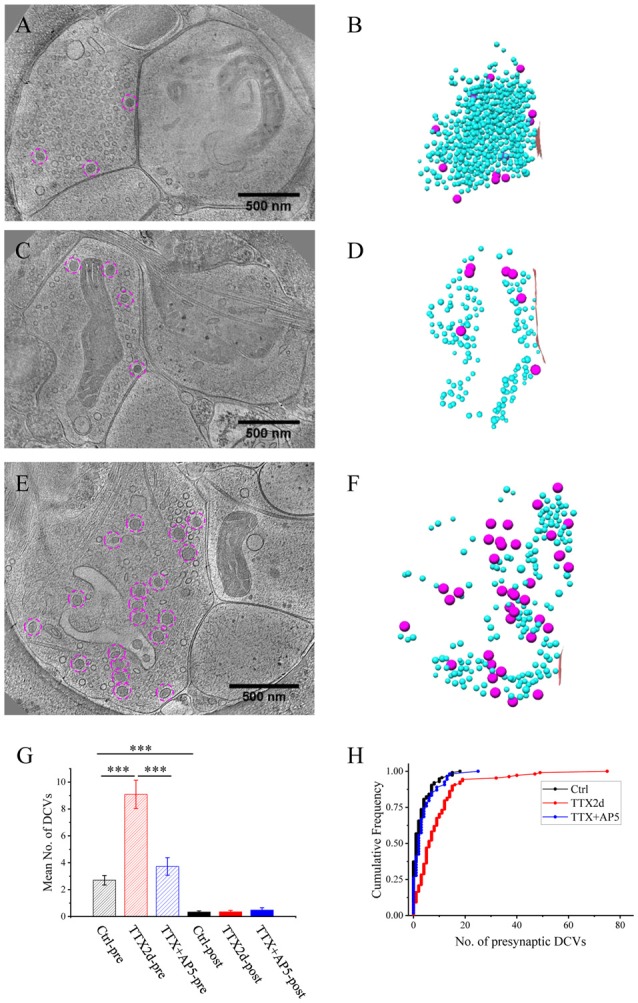
Accumulation of presynaptic DCVs in excitatory synapses after chronic inactivation. **(A,C,E)** Tomographic slices showing two spine synapses **(A,C)** and one shaft synapse **(E)** with 2-day tetrodotoxin (TTX) treatment. **(B,D,F)** 3D rendering of SVs (cyan ball), DCVs (magenta ball), and presynaptic membrane of active zone area (red) in the three synapses shown in **(A,C,E)**. **(G)** The mean number of DCVs in presynaptic boutons and postsynaptic compartments in synapses from the three groups: control (pre: 2.70 ± 0.36, post: 0.33 ± 0.08, *n* = 113 synapses, Mean ± standard error of the mean (SEM) hereafter), TTX 2d (pre: 9.08 ± 1.05, post: 0.36 ± 0.09, *n* = 107 synapses), and TTX 4 h + AP5 3 h (pre: 3.72 ± 0.65, post: 0.48 ± 0.17, *n* = 54 synapses). (Error bars are SEM. ***p < 0.001, two sample K-S test). **(H)** Cumulative histogram shows the number of presynaptic DCVs of the three groups.

**Figure 3:**
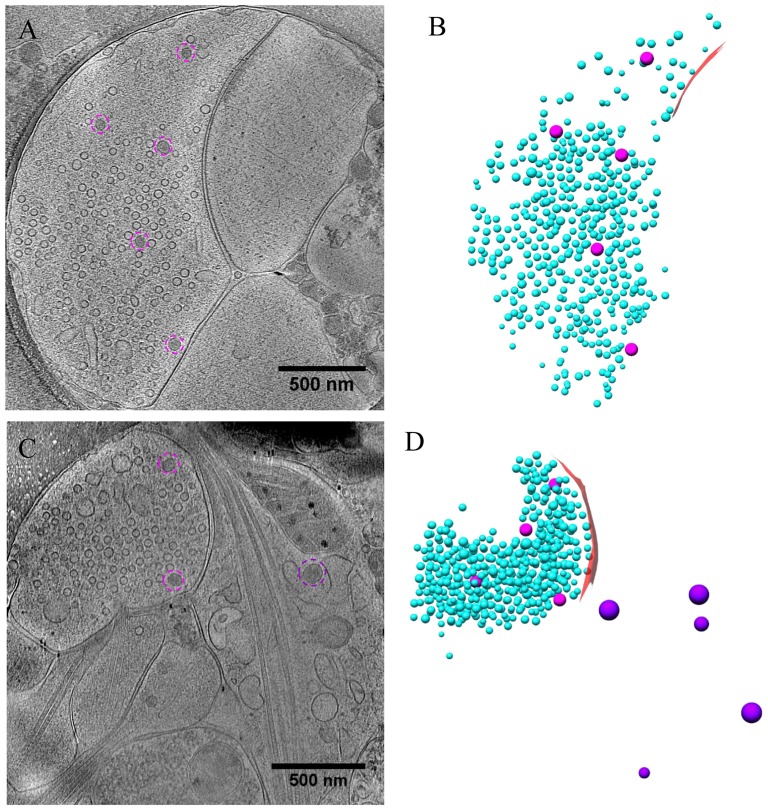
Ultrastructure of excitatory synapses after short-term inactivation. **(A,C)** Tomographic slices showing a spine synapse **(A)** and a shaft synapse **(C)** treated with TTX for 4 h and AP5 for 3 h. **(B,D)** 3D rendering of SVs (cyan ball), DCVs (magenta ball for presynaptic and purple ball for postsynaptic), and presynaptic membrane of active zone area (red) in the two synapses, respectively.

To visualize the ultrastucture of neuronal synapses in their native state, we cultured rat hippocampal neurons directly on gold electron microscopy (EM) grids, and cryo-fixed the sample by plunge freezing (Tao et al., 2018). Under cryo-ET, synapses in the vitrified samples were identified based on characteristic features, including pairs of apposed membranes with relatively uniform clefts between them, and vesicles of similar sizes on one side (Figures 1A–F). These synapses show marked heterogeneity in overall morphology and detailed ultrastructure. Among the 332 synapses we examined, the majority (274) were excitatory synapses (Figures 1–3), identifiable based on their distinct postsynaptic densities (PSDs; Colonnier, 1968; Peters and Palay, 1996; Tao et al., 2018), identified as a thick electron-dense layer attached to the postsynaptic membrane on its cytoplasmic side (Figures 1A,C,E). These synapses were formed on either dendritic spines (Figures 1A, 2A,E, 3A) or shafts (Figures 1C,E, 2C, 3C). Other structures inside synapses, including mitochondria, microtubules (MT), and a meshwork of actin filaments, were easily visualized in the tomograms (Figures 1A,C,E).

One of the most prominent features we observed in the presynaptic compartment was a population of vesicular structures, including small clear synaptic vesicles (SVs) of different sizes and shapes as characterized previously (Tao et al., 2018), and sometimes a few larger vesicles that had a higher luminal density (Figures 1A,C). These larger vesicles appeared to have contents different from those of regular SVs, and presumably corresponded to the DCVs observed in studies using conventional EM (Sorra et al., 2006). A scatter plot of the normalized luminal density against the diameter of all vesicular structures in four randomly chosen synapses shows two well-defined clusters, corresponding to SVs and DCVs classified with visual inspection (Figure 1G). Analysis of more synapses revealed distinct distributions of the sizes of SVs and DCVs (Figure 1H). The diameter of SVs ranged from 30 to 60 nm (Mean ± SD, 43.1 ± 4.6 nm, *n* = 930 from 4 synapses), consistent with previous studies (Tatsuoka and Reese, 1989; Fernández-Busnadiego et al., 2010; Harris and Weinberg, 2012). The diameter of DCVs ranged from 50 to 140 nm, (Mean ± SD, 80.1 ± 11.9 nm, *n* = 833 from 185 synapses), indicating that they were mostly small DCVs previously reported to be about 80 nm in size. It has been suggested that small DCVs may contain packs of active zone proteins such as Piccolo and Bassoon, and function as cargos for synaptogenesis in development (Ahmari et al., 2000; Zhai et al., 2001; Shapira et al., 2003; Sorra et al., 2006).

In the DIV16 cultures we examined, most excitatory synapses contained only 0–2 DCVs (75 out of 113 synapses) in their presynaptic boutons, and the other synapses had 3–18 presynaptic DCVs (Figures 1A–F, 2H). The mean number of presynaptic DCVs in our samples (2.70 ± 0.36, *n* = 113 synapses from eight batches of cultures; Figure 2G) is higher than that of rat hippocampal synapses *in vivo* (Sorra et al., 2006), perhaps reflecting the fact that at least some of these synapses in culture are still developing. We also observed some DCVs sparsely distributed in postsynaptic compartment (Figures 1E,F). These DCVs could contain neuropeptides such as BDNF, which can serve as a retrograde signal for activating presynaptic TrkB receptors to mediate presynaptic scaling (Jakawich et al., 2010). The number of postsynaptic DCVs (0.33 ± 0.08, *n* = 113 synapses) was significantly lower than that of presynaptic ones (*p* < 0.001, two sample Kolmogorov-Smirnov (K-S) test; Figure 2G).

### Accumulation of Presynaptic DCVs in Excitatory Synapses After Chronic Inactivation

We treated the cultured neurons with 1 μM TTX for 2 days, which causes upscaling of mature excitatory synapses (Turrigiano et al., 1998) but may also impair early synaptogenesis (van Huizen et al., 1985). A total of 107 excitatory synapses from seven batches of cultures were imaged and analyzed after 2 days of TTX treatment. The overall structure of these synapses experiencing chronic inactivity was similar to those without TTX treatment, all containing typical SV populations and PSD structures (Figures 2A–F). Interestingly, the number of presynaptic DCVs more than doubled with chronic inactivation (9.08 ± 1.05, *n* = 107 synapses, *p* < 0.001, two sample K-S test; Figure 2G). Notably, 6 of the 107 synapses contained a remarkably large number of presynaptic DCVs (more than 20; Figures 2E,F,H); this was never seen in the 113 control synapses. In contrast, postsynaptic DCVs remained scarce (0.36 ± 0.09, *n* = 107 synapses) after 2-day TTX treatment, similar to the control synapses (Figure 2G).

**Figure 4:**
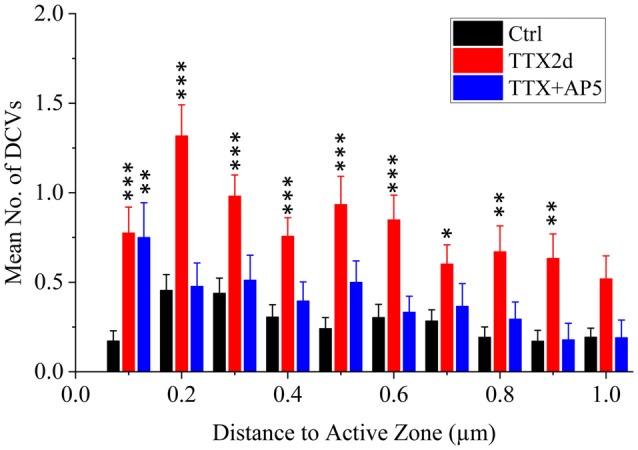
Mean number of presynaptic DCVs at different distances from the active zone area in excitatory synapses under different conditions. Comparisons were made between the inactivity treatments (either TTX2d or TTX+AP5) and the control. *p < 0.05; **p < 0.01; ***p < 0.001, Kruskal-Wallis ANOVA test.

As an alternative protocol, we blocked neuronal activity with TTX for 4 h together with AP5 for 3 h, a treatment that induces rapid upscaling of synaptic strength to a degree similar to that induced by chronic TTX treatment (Sutton et al., 2006). However, this treatment did not cause significant increase in the mean number of presynaptic DCVs (3.72 ± 0.65, *n* = 54 synapses from four batches of culture, compared to 2.70 ± 0.36; *p* = 0.289, two sample K-S test; Figures 2G,H, 3). Intriguingly, the number of DCVs near the active zone area (<100 nm) did show significant increase (Control: 0.17 ± 0.05, *n* = 99; TTX 2d: 0.78 ± 0.14, *n* = 107; TTX 4 h + APV 3 h: 0.75 ± 0.21, *n* = 33. Ctrl. vs. TTX 2d, *H* = 20, *χ*^2^ = 1, *p* < 0.001; Ctrl. vs. TTX+AP5, *H* = 9.8, *χ*^2^ = 1, *p* < 0.005, Kruskal-Wallis ANOVA test; Figure 4). Thus, short-term inactivity could also alter the organization of presynaptic DCVs, perhaps by blocking their release.

### Accumulation of Presynaptic DCVs in Inhibitory Synapses

**Figure 5:**
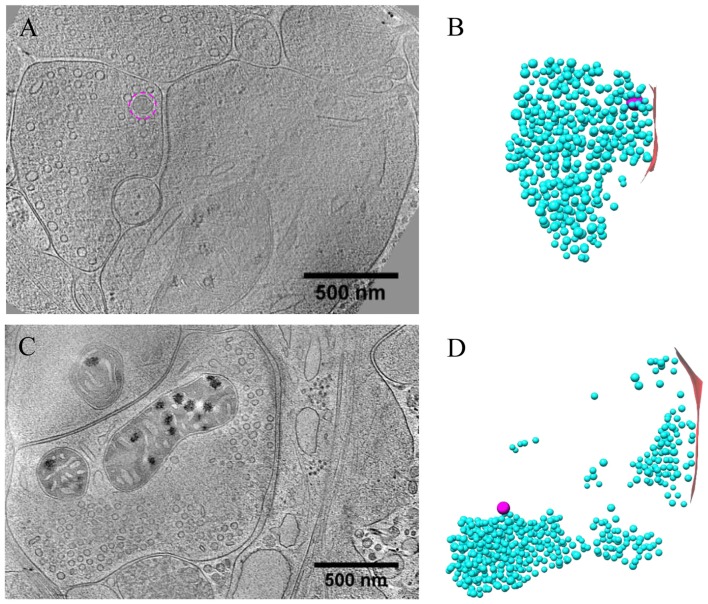
Ultrastructure of inhibitory synapses revealed by cryo-ET. **(A,C)** Tomographic slices showing two shaft synapses. **(B,D)** 3D rendering of SVs (cyan ball), DCVs (magenta ball), and presynaptic membrane of active zone area (red) in the two synapses, respectively.

**Figure 6:**
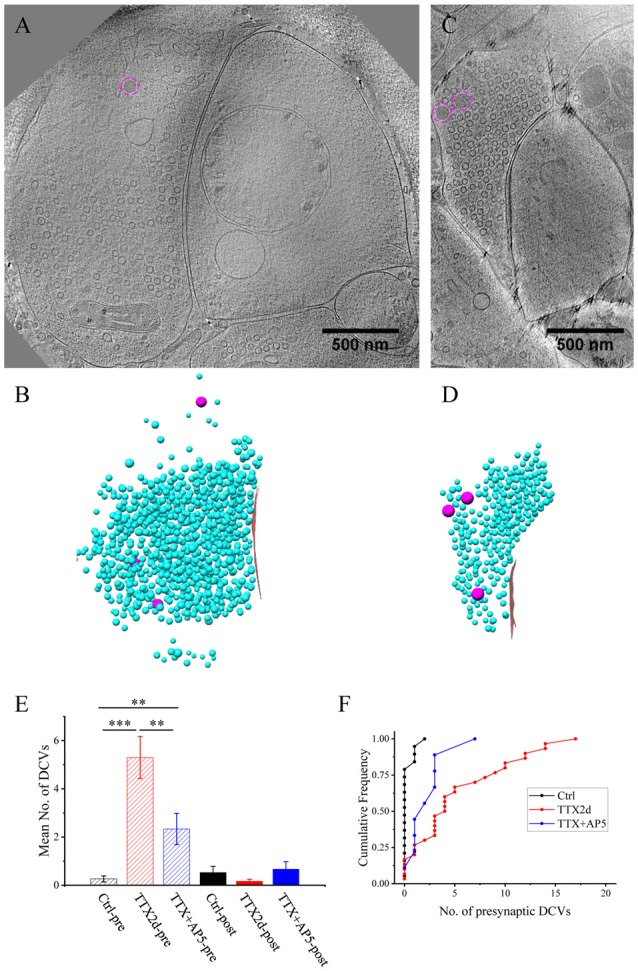
Accumulation of presynaptic DCVs in inhibitory synapses after chronic inactivation. **(A,C)** Tomographic slices showing two inhibitory spine synapses. **(B,D)** 3D rendering of SVs (cyan ball), DCVs (magenta ball), and presynaptic membrane of active zone area (red) in the two synapses, respectively. **(E)** The mean number of DCVs in presynaptic boutons and postsynaptic compartments in synapses from control group (pre: 0.26 ± 0.12, post: 0.52 ± 0.25, *n* = 19 synapses), TTX 2 days (pre: 5.30 ± 0.87, post: 0.17 ± 0.08, *n* = 30 synapses), and TTX 4 h + AP5 3 h (pre: 2.33 ± 0.65, post: 0.67 ± 0.31, *n* = 9 synapses). (**p < 0.01, ***p < 0.001, two sample K-S test). **(F)** Cumulative histogram shows the number of presynaptic DCVs of the three groups.

**Figure 7:**
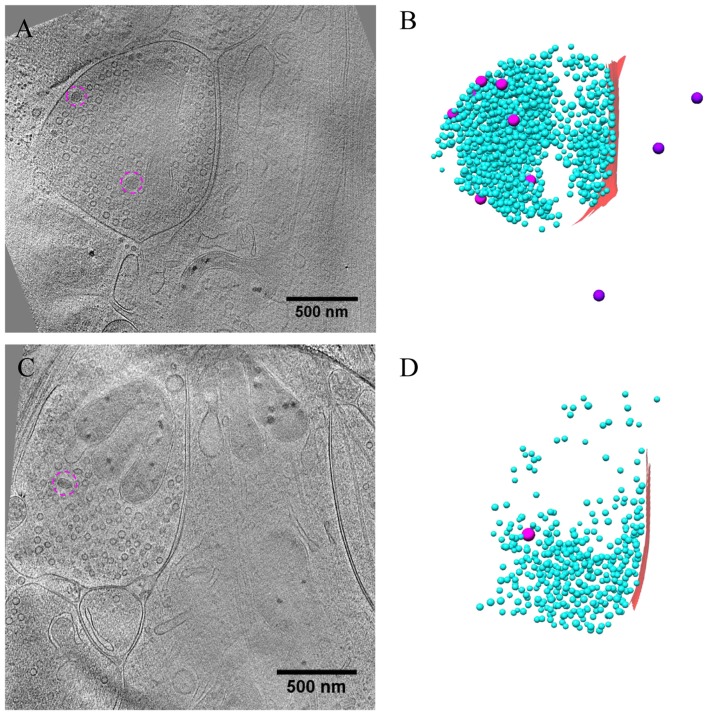
Ultrastructure of inhibitory synapses after short-term inactivation. **(A,C)** Tomographic slices showing two shaft synapses treated with TTX for 4 h and AP5 for 3 h. **(B,D)** 3D rendering of SVs (cyan ball), DCVs (magenta ball for presynaptic and purple ball for postsynaptic), and presynaptic membrane of active zone area (red) in the two synapses, respectively.

Besides the majority of excitatory synapses, we also obtained inhibitory synapses, primarily GABAergic synapses in the hippocampal cultures, under different conditions (Figures 5–7). The identification of inhibitory synapses was based on their uniform thin sheet-like PSD structures as characterized using correlative microscopy in our previous work (Tao et al., 2018). The majority of inhibitory synapses (40 out of 58) contained MT in their postsynaptic compartments (Figures 5A,C, 7A,C), consistent with previous observations that most inhibitory synapses were located on dendritic shafts (Harris and Weinberg, 2012).

Under control conditions, the number of presynaptic DCVs in inhibitory synapses (0.26 ± 0.12, *n* = 19 synapses from eight batches of cultures) was much lower than that in excitatory synapses (*p* < 0.005, two sample K-S test). Yet, after chronic inactivity, the inhibitory synapses showed marked accumulation of presynaptic DCVs (5.30 ± 0.87, *n* = 30 synapses from five batches of cultures, *p* < 0.001, two sample K-S test; Figures 6E,F). Notably, the number of presynaptic DCVs also increased significantly with short-term inactivation by TTX 4 h and AP5 3 h (2.33 ± 0.65, *n* = 9 synapses from four batches of cultures, *p* < 0.01, two sample K-S test; Figures 6E,F). Due to limited data for inhibitory synapses, we were not able to analyze the distribution of their presynaptic DCVs as a function of distance from the active zone.

### Release of DCVs at Active Zone and Peri-Active Zone Sites

**Figure 8:**
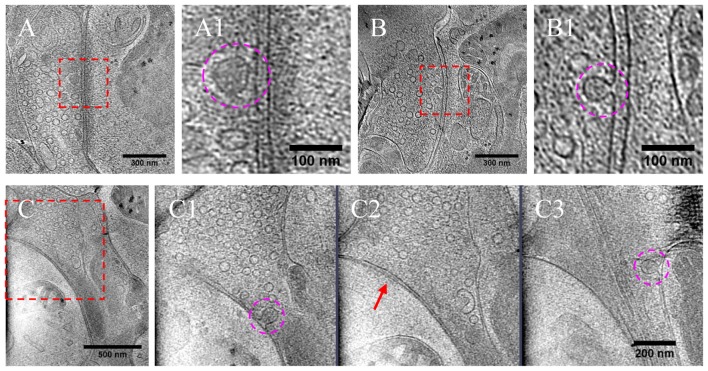
DCVs docked or fused at active zone and non-active zone areas. **(A)** Tomographic slice showing one DCV docked in active zone site, indicated with magenta circles in the zoomed-in view **(A1)**. **(B)** One DCV undergoing fusion in the active zone site, indicated with magenta circles in the zoomed-in view **(B1)**. **(C)** Tomographic slice showing one synapse with DCVs released in non-active zone site. **(C1–C3)** Three zoomed-in views of the boxed area in **(C)**, but of different virtual sections (z coordinates). **(C1)** One DCV (magenta circle) fused with presynaptic membrane at peri-active zone site. **(C2)** The synaptic junction area is indicated with a red arrow. **(C3)** One DCV (magenta circle) fused at opposed site of the active zone.

As most DCVs are small DCVs thought to carry protein cargos for presynaptic active zone assembly (Ahmari et al., 2000; Zhai et al., 2001; Shapira et al., 2003; Dresbach et al., 2006), we investigated whether DCVs could be detected in a docked or partially fused state. Indeed, we observed docked DCVs within 20 nm from the presynaptic active zone membrane, with tethers found by visual inspection connecting the vesicle to the membrane (Figures 8A,A1), as well as partially fused DCVs at the active zone or peri-active zone areas (Figures 8B,B1,C–C3). Interestingly, there were many more docked DCVs, especially in excitatory synapses, under both chronic and short-term inactivation conditions, compared to the control condition (Table 1). These results are consistent with our speculation that inactivity blocked DCV release, thus causing accumulation at different stages of DCV trafficking.

**Table 1:** Number of synapses with docked or fused dense core vesicles (DCVs) in different condition.

	Control		TTX (2d)		TTX (4 h) + AP5 (3 h)	
	Excit.	Inhi.	Excit.	Inhi.	Excit.	Inhi.
No. of synapses examined	113	19	107	30	54	9
DCVs docked at AZ*	5/3	0	49/23	1/1	7/5	0
DCVs docked at peri-AZ*	3/3	0	6/4	0	0	0
DCVs fused at AZ*	0	0	1/1	2/2	1/1	0
DCVs fused at peri-AZ*	0	0	0	1/1	0	0

*Data are shown as No. of DCVs/No. synapses containing the DCVs. Excit.: excitatory synapses; Inhi.: inhibitory synapse.

In a few cases (Figures 8B,B1,C–C3, Table 1), DCVs were captured that appeared to be undergoing fusion with the presynaptic membrane, generating an omega-shaped structure with a narrow neck. The luminal density of the fusing DCV was still evident, suggesting that the release of the inner content of DCV is slow compared to the formation of the omega-shaped structure. This phenomenon also indicates that kiss-and-run release of the DCV, if occurs, may not be sufficient to deplete its contents.

## Discussion

The majority of presynaptic DCVs we observed can be classified as “small DCVs” based on their ~80 nm diameter; small DCVs are believed to carry active zone components important for the construction of synapses (Vaughn, 1989; Sorra et al., 2006). It is not surprising to observe these DCVs in cultured hippocampal neurons of DIV16, a stage at which many synapses are still developing. The increase in the number of presynaptic DCVs upon chronic blockade of neuronal activity is likely due to accumulation of DCVs transported to the synapse but unable to release. Unlike SVs that can spontaneously fuse to the presynaptic membrane in the absence of action potentials, these presynaptic DCVs may require a higher level of activity or calcium to release. Indeed, it is known that DCV release may be related to ryanodine receptor-mediated calcium store release (Hoover et al., 2014; Nurrish, 2014). Intriguingly, postsynaptic DCVs did not accumulate during inactivity, suggesting that the two types of DCVs might use different sets of trafficking/exocytosis machinery.

From the result of 2-day TTX treatment, an average excitatory synapse contained ~9 DCVs in the presynaptic bouton, ~6 more than the average control bouton. Meanwhile, an average inhibitory synapse after 2-day TTX treatment contained ~5 DCVs more than control. This could suggest that with activity blockade, DCVs in both types of synapses accumulated within the presynaptic bouton at a rate of ~1 DCV every 8 h (7.5 ± 1.3 h per DCV for excitatory synapses; 9.5 ± 1.7 h per DCV for inhibitory synapses). Accumulation of presynaptic DCVs has also been observed in developing optic tectal synapses in *Xenopus laevis* following visual deprivation (Li and Cline, 2010), indicating that DCV accumulation in synaptic boutons could be a ubiquitous process in response to inactivity. These observations are consistent with a role of DCV in normal trafficking of cargo proteins to the active zone and perhaps also the synaptic cleft in developing synapses (Ahmari et al., 2000; Zhai et al., 2001; Shapira et al., 2003). Under control conditions, an inhibitory synapse contains on average 0.3 presynaptic DCVs, whereas an excitatory synapse contains 2.7 DCVs. If we assume that the rate of DCV accumulation during inactivity is equivalent to the replenishment rate under normal steady-state conditions without activity block, these numbers may reflect a higher release probability of DCVs in normal inhibitory synapse. This could mean that inhibitory synapses are more mature in these cultures. Meanwhile, short-term inactivation (TTX 4 h + AP5 3 h) resulted in significant increase of presynaptic DCVs in inhibitory synapses but not excitatory synapses, suggesting that inhibitory synapses may have an additional fast recruitment mechanism upon inactivation. Interestingly, it is also known that inhibitory synapses exhibit much faster structural turnover *in vivo* (Villa et al., 2016).

How might the inhibition of DCV release, or the accumulation of DCVs by activity blockade, play any role in synaptic development and homeostasis? One possibility is that with activity blockade, the cargo proteins, e.g., active zone components, cannot be delivered to the target location, thus delaying the maturation of the synapse. Indeed, activity is known to be required for synapse maturation (Bergey et al., 1981; Janka and Jones, 1982; Li and Cline, 2010). It is also possible that the DCVs may carry signaling and scaffolding molecules that can elicit pre- or postsynaptic responses during functioning of the synapse. The lack of these signals may cause compensatory responses at the synapse, resulting in enhancement of synaptic efficacy. Such a “negative signaling” scheme has been proposed for CaMKIV to detect reduction of activity and to mediate synaptic scaling (Ibata et al., 2008; Goold and Nicoll, 2010). Whether the first or the second mechanism wins may depend on conditions of the synapses such as their level of maturity.

As in other forms of calcium-regulated vesicular transport and release (Bi et al., 1997), DCV trafficking is likely to be regulated at multiple stages. Indeed, in a reconstituted system, calcium increases the probability of both docking and fusion of DCVs (Kreutzberger et al., 2017). Following chronic inactivity, more DCVs accumulated in the presynaptic compartment, and arrested in docking states. These DCVs could have high probability to be released upon reactivation of the neurons, especially by the elevated level of calcium during the repetitive stimulation required for the induction of LTP. Such elevated DCV release, due to 9-fold increase in docked DCVs (Table 1), 4-fold increase in recruited DCVs to near-active zone area (Figure 4), as well as 2-fold increase in overall presynaptic DCVs (Figure 2G), potentially provides signaling and structural material for extra synaptic strengthening. Note that among chronically inactivated synapses, the majority of synapses (84 out of 107) did not exhibit docking DCVs, while 49 DCVs docked in 23 synapses. Thus, perhaps only a small portion of synapses could exhibit strong “hyper-potentiation” upon reactivation. Nevertheless, this scenario may explain the observed enhancement of LTP induction in hippocampal neurons following chronic TTX treatment (Gerkin et al., 2013). Of particular interest is that a significant portion of the enhanced LTP persisted in the presence of AP5 that suppressed normal NMDAR-dependent LTP (Gerkin et al., 2013). Such NMDAR-independent extra LTP is likely to be expressed presynaptically, perhaps mediated by the fusion of reserved DCVs. Thus, the accumulated DCVs may serve as a reserve resource for synaptic metaplasticity.

## Author Contributions

C-LT, Y-TL, P-ML, ZHZ and G-QB conceived and designed the experiments. C-LT and Y-TL performed the experiments. C-LT, Y-TL and G-QB analyzed the data and wrote the article. All authors reviewed and approved the article.

## Conflict of Interest Statement

The authors declare that the research was conducted in the absence of any commercial or financial relationships that could be construed as a potential conflict of interest.
